# From intersubjective correspondence to the counterpart relation—motifs from Carnap’s Aufbau in Lewis’s counterpart theory and his philosophical methodology

**DOI:** 10.1007/s44204-026-00384-1

**Published:** 2026-02-17

**Authors:** Robert Michels

**Affiliations:** LanCog, CFUL, University of Lisbon, Lisbon, Portugal

**Keywords:** Rudolf Carnap, David Lewis, Der logische Aufbau der Welt, The logical construction of the world, Counterpart theory, Intersubjective correspondence

## Abstract

In ‘Counterpart Theory and Quantified Modal Logic’, David Lewis remarks that his counterpart relation ‘is very like’ the relation of intersubjective correspondence in Rudolf Carnap’s ‘Der logische Aufbau der Welt’. Given Lewis’s status as the most eminent contemporary metaphysician, and Carnap’s as the most eminent twentieth century critic of metaphysics, this reference may not only be surprising, but it is also suggestively opaque. In which respect could the two relations from two wildly distinct philosophical frameworks resemble each other? The main aim of this paper is to argue that Lewis’s claim indeed points to a significant similarity: both relations act as replacements for identity in their respective frameworks. This main argument is supplemented by a discussion of three further similarities between aspects of Lewis’s methodology and Carnap’s methodology of the Aufbau. The paper hence supports recent efforts to show that Carnap’s book was not the philosophical misstep it was taken to be by Quine and Goodman, but that it rather should be seen as an important step in the development of analytic philosophy.

## A surprising footnote

In his early paper ‘Counterpart Theory and Quantified Modal Logic’ (Lewis, 1968), David Lewis introduces counterpart theory as an alternative to quantified modal logic. The paper contains the following passage:

The counterpart relation is a relation of similarity. So it is problematic in the way all relations of similarity are: it is the resultant of similarities and dissimilarities in a multitude of respects, weighted by the importances of the various respects and by the degrees of the similarities. (Lewis (1968), p. 115.)

While this passage is interesting in itself,^1^ my focus here will rather be on Lewis’s footnote to its last sentence^2^:

The counterpart relation is very like the relation of intersubjective correspondence discussed in Rudolf Carnap, Der Logische Aufbau der Welt (Berlin-Schlactensee[sic!]): Weltkreis-Verlag, 1928), sec. 146. (Lewis (1968), p. 115.)

That Lewis refers to Carnap is not unusual^3^, and he even wrote a paper specifically on the Aufbau, ‘Policing the Aufbau’, which proposes a solution to an important technical problem in Carnap’s book, the problem of imperfect community.^4^

What makes the footnote noteworthy is the stark contrast between Lewis’s likeness claim about the two relations and the striking dissimilarity between the two different contexts in which these relations do their theoretical work. The counterpart relation is a component of Lewis’s unapologetically metaphysical theory of modality, a theory which was already partially present in Lewis’s early papers. In Lewis (1968), the counterpart relation is introduced as an axiomatically defined predicate of classical first-order logic without constants which permits us to formalize modal talk without using modal operators. The counterpart relation is a relation of overall similarity which relates an object from one world to distinct objects in other possible worlds, where each object is world-bound, i.e. each only exists in one possible world.^5^ In the context of this formal framework, the relation is Lewis’s ‘substitute for identity between things in different worlds’ (Lewis (1968), p. 114; see Section 3.1 for a more detailed introduction of the relation).

The relation of intersubjective correspondence in contrast is a component of the constructional system sketched in Carnap’s *Aufbau*,^6^ a system of definitions which provides a rational reconstruction of all scientific knowledge on an autopsychological basis, i.e. on the basis of the basic experiences of a hypothetical single subject which undertakes the reconstruction. The definitions making up this system are hierarchically ordered in four levels: the autopsychological, physical, heteropsychological, and cultural. As part of this construction, analogous constructional systems for other persons are introduced on the heteropsychological level of the main system (i.e. they are ultimately still constructed out of the basic experiences of the subject of the main system), leading to a proliferation of different ‘subjective’ versions of the same objects on the physical levels of these sub-systems and the main system. It is there that the relation of intersubjective correspondence is introduced in order to construct ‘the intersubjective world’ (Carnap (1969), §148) of physics. It captures that ‘the spatiotemporal relations which hold for the physical world points’ on the physical level of a sub-system also ‘hold for the corresponding world points’ (Carnap (1969), §146) on the physical level of the original system. I will give more context and introduce the relation more fully in Section 3.2. In the current context, I want to stress that Carnap’s sketched constructional system illustrates a fundamental methodological approach to philosophy, constructional theory. In the last part of the *Aufbau* (Carnap (1969), part V), this approach is explicitly used to further the anti-metaphysical agenda which Carnap pursued at the time of the publication of the Aufbau, and which he brought to its apex soon afterwards (see Carnap (1959)).^7^

The two relations stem from very different, and in many ways, conflicting philosophical projects. This raises the question of whether there is any reason to think that Lewis’s likeness claim may hint at a substantive similarity. In this paper, I argue that this is indeed the case, supporting (MacBride, 2021)’s recent argument that there is a relation of *ancestry* between some ideas in Carnap’s Aufbau and in Lewis’s modal metaphysics (meaning that there is a systematic relation between the ideas).^8^ The paper hence contributes to the broader historical project of re-evaluating the historical role played by the Aufbau in contemporary analytic philosophy, questioning the still prevalent narrative reaching back to Lewis’s teachers Quine and Goodman, that the Aufbau was a philosophical failure. If methods and ideas from the Aufbau reoccur in a very different theoretical context, then this indicates that, rather than being a mere historical curiosity, the book played an anticipatory role in the history of analytic philosophy. This is especially salient in this case, since Lewis was one of the most influential analytic philosophers of the twentieth century, who set the agenda in a number of different discussions, particularly in, but not limited to metaphysics.^9^

Does Lewis’s reference indicate more than a historical ancestry? Did the Aufbau perhaps play a causal role in the development of counterpart theory? To the best of my knowledge, Lewis himself did not address this question, but there is some relevant historical evidence. Lewis likely first engaged with the Aufbau when he took a class with Goodman on his ‘The Structure of Appearance’ (Goodman, 1951) at Brandeis in Spring 1965.^10^ In October of the same year, Lewis already deploys counterpart theory in a letter to Shaffer (Lewis (2020), letter 120). In a letter to Føllesdal in March of the following year (Lewis (2020), letter 121), Lewis explicitly mentioned Hintikka, but not Carnap, as an influence on counterpart theory. His work on the theory can furthermore be tracked back to 1963. (See Beebee and Fisher (2022), pp. xvii-xviii.) While this timeline does not rule out an influence on the development of counterpart theory, it at least makes it seem unlikely that Lewis only came up with the counterpart relation after reading the Aufbau. I will not further discuss this historical question in what follows, but will rather focus on the question of whether Lewis’s similarity claim indeed points to a noteworthy parallel to the Aufbau.

To clarify Lewis’s likeness claim, I will present and discuss two different readings of the claim. The first is a purely formal reading, which might at first appear plausible, but turns out to be untenable (Section 2). I then introduce the second reading, which interprets Lewis’s claim as a claim about the theoretical roles played by the two relations. As I will show, this reading points to a substantial similarity: both relations are substitutes for identity in their respective contexts (Section 3). My main point will be that this reading gives us an answer to the main question of the paper: so understood, his claim is plausibly true, pointing to the mentioned ancestry of ideas. In Section 4, I discuss a potential problem for the preceding main argument related to the placement of Lewis’s footnote. Section 5 concludes the paper with a discussion of three respects in which Lewis’s philosophy resembles the Aufbau on a methodological level: first, Lewis’s reliance on quasi-analysis in a proposed version of nominalism in Lewis (1983); second, Lewis’s own reflections on the continuity of his paper on the Aufbau and his work in the philosophy of mathematics; and third and finally, a discussion of the parallels between the theoretical use to which a notion of overall similarity is put in the Aufbau and in Lewis’s philosophy. This last section hence shows that the similarities in Lewis’s philosophy to Carnap’s early book are more ubiquitous and run even deeper than one might think.

## The purely formal reading

What did Lewis mean when he wrote that the counterpart relation is ‘very like’ the relation of intersubjective correspondence? According to the first reading of this claim which I want to discuss, he was referring to a similarity regarding the formal properties of the two relations, indicating that they are isomorphic, or to use Carnap’s term from the Aufbau, structurally equivalent (see Carnap (1969), §§11-12).

The formal properties of a relation are properties which can be characterized set-theoretically based solely on its extension. Since both Carnap in the Aufbau and Lewis in Lewis (1968) assume a strictly extensionalist standpoint, this reading is a natural starting point for an interpretation of Lewis’s likeness claim.^11^

If correct, this reading would immediately defuse the felt tension which makes Lewis’s claim appear surprising, since this tension derives from the material (i.e. non-formal) meaning the two relations are associated with in their respective theoretical contexts. The footnote would then state a correct but perhaps unremarkable fact, dashing anyone’s hope that the reference might reveal something of genuine interest about the philosophical ancestry of Lewis’s counterpart theory.

### The formal properties of the counterpart relation

In the following, I will focus on three particularly important formal properties, reflexivity, symmetry, and transitivity, starting with Lewis’s relation. That the counterpart relation is reflexive immediately follows from Lewis’s postulate P6 in Lewis (1968), which says that anything in a world is a counterpart of itself, matching the fact that everything is overall similar to itself. (See Lewis (1968), p. 114.) The two other formal properties of the counterpart relation are determined by its status as a relation of overall similarity. Like any relation of this kind, the counterpart relation is neither symmetric nor transitive. (See Lewis (1968), p. 116.)

### The formal properties of the relation of intersubjective correspondence

What are the formal properties of the relation of intersubjective correspondence? The relation is introduced on the third, the heteropsychological level of Carnap’s outlined constructional system in Part IV of the Aufbau. Unlike in the first, the autopsychological level, the construction is at this point no longer carried out explicitly, but merely sketched, and Carnap also no longer explicitly states the formal properties of all the relations he introduces. The only formal property explicitly mentioned in the paragraphs dedicated to the discussion of intersubjective correspondence (§§146–148; citations by paragraph without source are always to the Aufbau) is that it is a ‘one-to-one correspondence’ (‘eineindeutige Zuordnung’, §146) in the following sense: if we take the main system and one of its sub-systems constructed for another subject (where this sub-system is still entirely constructed out of the subject of the main system’s experience), then each object at the physical level of one of these two systems intersubjectively corresponds to exactly one object on the physical level of the other (cf. §146). Since these systems are constructed based on the basic experiences of different subjects, one can, speaking not in proper terms of the actual formal construction, but in terms of the empirical sciences,^12^ say that ‘[t]wo intersubjectively corresponding objects [...] represent “the same” object, once as it is recognized by me [i.e. the subject on whose basic experiences the main system is based] and the other time as it is (so far as I know) recognized by M [another subject on whose basic experience, as represented in the main system, the sub-system is based]’ (§146.). This indicates that intersubjective correspondence sorts objects from a constructional system and its sub-systems into equivalence classes.

A bit of backtracking in the text confirms this picture: In §148, Carnap introduces the intersubjective objects as the abstraction classes of the relation of intersubjective correspondence, referencing §73, which is specifically concerned with abstraction classes formed based on equivalence relations. Intersubjective correspondence is hence an equivalence relation. Like any equivalence relation, intersubjective correspondence is reflexive, symmetric, and transitive.

### The purely formal reading: verdict

We have seen that the counterpart relation is reflexive, but neither symmetric nor transitive, whereas the relation of intersubjective correspondence is an equivalence relation, i.e. reflexive, symmetric, and transitive. While there are of course further formal properties one might consider, the properties discussed here are central enough to allow us to conclude that, regarding their purely formal properties, the two relations are not ‘very like’, their shared reflexivity notwithstanding. This is a sufficient reason to dismiss this first reading.

## The theoretical role reading

The core idea of this subsection is that the most fundamental role played by both the counterpart relation in Lewis’s modal metaphysics and the relation of intersubjective correspondence in Carnap’s outlined phenomenalist constructional system is that of being a substitute for identity. Both relations hold between objects which can, for reasons specific to the respective theoretical frameworks, not be identical. An identity-like relation between such objects is nonetheless needed in both cases, also for framework-specific reasons.

To argue that both relations play this role in their respective theoretical contexts, I will, for each of the two relations, answer the following questions: Why can the objects which stand in the relevant relation not be identical in the relevant theoretical context? And why is an identity-like relation still needed?

### The counterpart relation as a substitute for identity

A crucial assumption of Lewis’s counterpart theory is that material objects are world-bound in the sense that they are wholly located in one single possible world. This assumption allows Lewis to avoid notoriously thorny issues in modal metaphysics and modal logic which are tied to the notion of trans-world identity.^13^

His strategy is simply to deny that there are trans-world identical objects: ‘Within any one world, things of every category are individuated just as they are in the actual world; things in different worlds are never identical[...]’.^14^ That the same thing exists in different possible worlds (trans-world identity) is hence ruled out by fiat, cutting any metaphysical problem this could lead to at the root.

Lewis’s commitment to world-bound objects however raises a question about the treatment of de re modality: Given that all objects are word-bound, how can one account for the truth of de re modal claims such as ‘The Eiffel Tower could have been painted green’? After all, if the Eiffel Tower only exists in the actual world, there is no possible world in which *it* is, contrary to actual fact, green.

Lewis’s answer to this question builds on the counterpart relation. While there is no merely possible world in which the Eiffel Tower itself is painted green, there is a world in which one of its *counterparts*, a distinct object which more closely resembles the Eiffel Tower than any other object does in that world, exists and is painted green. According to Lewis, the presence of this counterpart ensures that this world *represents the Eiffel Tower as being painted green*, rendering the de re modal claim in question true. Lewis’s idea is hence that the counterpart relation, a binary relation which holds between an object and the object(s) in a different possible world which resemble it more closely than any other object in that world,^15^ can act as a stand-in for the relation of (trans-world) identity: ‘[t]he counterpart relation is our substitute for identity between things in different worlds’. (Lewis (1968), p. 114.) As such, it does what this relation is otherwise supposed to do in the context of a semantic account of de re modal statements.

### The relation of intersubjective correspondence as a substitute for identity

To answer the two guiding questions (to repeat: Why can the objects which stand in the relevant relation not be identical in the relevant theoretical context? Why is an identity-like relation still needed?), I will first more fully introduce the relation of intersubjective correspondence and elaborate its theoretical role (showing why an identity-like relation is needed at the relevant point in the Aufbau and answering the second question). Then I will clarify what identity amounts to in the context of constructional theory and show why intersubjectively corresponding objects cannot be identical in that sense (answering the first question).

#### Intersubjective correspondence in Carnap’s example system

In part IV of the Aufbau, Carnap partly elaborates an example of a constructional system with a phenomenalistic basis. In general, constructional systems are systems of definitions which make use of a number of primitive non-logical relations plus logical notions in order to produce a rational reconstruction of the whole of science. In case of Carnap’s example system, this reconstruction has a discrete hierarchical structure which reflects relations of epistemic fundamentality. (See §54.) It has four layers (in order of fundamentality): the autopsychological, physical, heteropsychological, and the cultural level. This hierarchy is built up based on a single primitive relation, the relation of *recollection of similarity*, *Rs*, whose relata are unstructured time slices of the phenomenal experience of a single subject. Given this solipsistic basis, one of the main problems which Carnap faces is to account for the objectivity of scientific knowledge. The relation of intersubjective correspondence plays a crucial role in Carnap’s attempt at a solution.

Carnap’s basic idea is that within this system, objectivity can be achieved by a process of constructional bootstrapping: the hypothetical subject carrying out the construction first constructs the physical world from its own subjective experience, then it uses the elements of the physical world to construct, first, other minds, then culture and artefacts, giving us a (save for gaps due to epistemic limitations) complete, but in certain respects inherently subjective reconstruction of the world. Drawing both on what it knows about other minds and the reports of other people, the subject then fleshes out this reconstruction, adding a structurally similar and likewise subjective reconstruction (also in the form of a constructional system, but this time a sub-system of the main system) of the world for each other subject on the heteropsychological level of its own reconstruction. All of course while still ultimately drawing only on its own experiences in the guise of the only non-logical primitive of the constructional system, the relation *Rs*. (See §§144,145.) Objectivity, which in this context amounts to a form of intersubjectivity, is then attained by stripping away all the subjective features which are specific to any of the constructional systems, retaining only the structure common to all of them.

To understand how the relation of intersubjective correspondence helps in doing this, we need to take a closer look at the structure of the heteropsychological level of a constructional system. As just stated, this level contains constructions of the worlds of other persons in the form of constructional systems built up on their (constructed) phenomenal experiences within the main constructional system, in the form of constructional sub-systems. The lowest levels of each such system, its respective autopsychological level, have the exact same logical structure as that of the main system. (See §121.) Everything on this level is constructed, that is defined, in the exact same manner, using the same definitions, where these definitions only contain logical vocabulary plus, and this is the only difference between them, the basic relation *Rs* of the respective (sub-)system, i.e. the particular recollections of similarity of the subject for which this sub-system is constructed. Notions defined on this level include similarity circles, quality classes (the constructional analogue to properties), the visual field, and the colour solid.

Beyond this first level, the construction involves empirical elements which are specific to the particular constructional (sub-)system. The construction of the second level, the physical level, starts with an assignment of colours from the colour solid to world points, i.e. with the definition of a mosaic of colour spots (think of the pixels of a computer screen) distributed over an abstract geometrical spacetime, representing the subject’s visual field. This assignment reflects the visual perspective of the constructing subject, starting with the construction of its points of view, i.e. the vantage point from which the subject sees the colour points of which its visual world consists, at a time. (§§126,127.)

At this stage, the analogy in the form of the construction between different (sub-)systems is still there, but only to a certain extent. Carnap gives the construction of ‘my body’ in different systems as an example. Within the main system *S*, this constructed object is the body of the subject of *S*. In a constructional sub-system *S*$_M$ constructed for a different subject *M* on the heteropsychological level of *S*, ‘my body’ is in contrast *M*’s body. Both instances of ‘my body’ will still share certain formal properties in virtue of being constructed following, so to say, the same plan.

For example, as they are physical things, they are both first constructed as visual things, i.e. as classes containing tuples of tuples consisting of three space and one time coordinate plus a visual quality. In both cases, these constructions are made based on the visual perspective of the subject of the respective (sub-)system: In *S*, the body of the constructional subject of *S* is first constructed as a visual thing consisting of distributions of colour spots at a time which are always very close to the point of view of the subject of *S*, are never completely seen by it, move in coordination with other sense qualities (e.g. tactile qualities) perceived by the subject, etc.. (See §129.) In case of *M*’s body in *S*$_M$, it is always very close to *M*, never completely seen by *M*, moves in coordination with other sense qualities perceived by *M*, etc. The particular classes of colour spots, and with them the constructed visual objects, will hence differ for the body of the subject of *S* in *S* and for *M*’s body in *S*$_M$ due to the perspectival nature of how ‘my body’ is constructed.

This illustrates how objects on the physical level of distinct constructional (sub-)systems differ in general: the ego-centric perspective of the subject for whom the system is constructed is reflected in the construction of each object on the physical, the heteropsychological, and the cultural level. The reconstruction of the whole physical world we find in *S* and those we find in any of its constructional (sub-)systems are populated by, so to say, differently defined versions of what, from a perspective outside these systems, which at this stage of the construction is not yet present in *S*, are the same physical objects.

To move from this multitude of (sub-)system-specific perspectival variants of an object to the single, perspective-independent objects of science, Carnap relies on the relation of intersubjective correspondence:

A one-to-one correspondence holds between the spatiotemporal world of physics in *S* and that in *S*$_M$ in the following way: the spatiotemporal relations which hold for the physical world points in *S*$_M$ also hold for the corresponding world points in *S*. The same is true for qualitative relations (i.e. relations which hold on the basis of assignment). For reasons to be explained later, we wish to call this correspondence *intersubjective correspondence*. (§146.)

So for example *M*’s body is constructed as the body of another person in *S*, but as *M*’s own body in *S*$_M$ (M’s constructional sub-system). Assuming for the moment that Carnap’s plan to draw the objective world of physics out of the subjective hat of the subject of *S*’s unstructured experiences works out, the two objects still have the exact same properties on the physical levels of *S* and *S*$_M$ respectively, where these properties include their visual, tactile, and auditory properties.^16^ The two objects intersubjectively correspond to each other in virtue of being indistinguishable regarding the physical relations they stand in and, derivatively, given the precedence of relational structure in constructional theory (see §§10-12), also regarding their physical properties. Carnap’s idea is that this correspondence can be extended to any sub-system constructed for a subject on the heteropsychological level of *S* and that a physical object (in the ordinary, not in the specific sense of constructional theory) is the abstraction class of all intersubjectively corresponding objects throughout all constructional (sub-)systems, i.e. *S*’s system and all sub-systems constructed within it on its heteropsychological level. In the context of Carnap’s example of a constructional system with a phenomenal basis, the relation of intersubjective correspondence is hence needed to construct the intersubjective, that is, the objective physical world of science. (Cf. §§148-9.) This answers the second guiding question of this section.

#### Identity and intersubjective correspondence in the Aufbau

Having specified the role which intersubjective correspondence plays in Carnap’s example of a constructional system, it still needs to be shown that the relation acts as a replacement for identity, because identity itself is not available to achieve a particular aim. It should be clear by now what that aim is: to define the objective world of science within a constructional system with a phenomenalistic basis, departing from a multitude of different, partly ego-centric constructional systems consisting of the main system and its sub-systems. To make the remaining point, let me start by getting clear about what ‘identity’ means in the context of the Aufbau.

As expected, the notion of identity in the Aufbau is directly tied to that of an object. The constructional conception of objects however is rather unusual. Objects in a constructional system are *quasi objects*, which are not really objects in the ordinary sense of the word, but rather the extensions of concepts. As Carnap puts it, ‘in construction theory we sometimes speak of constructed objects, sometimes of constructed concepts, without differentiating’. (§5.) ‘Concept’ here has a Fregean meaning (see Frege (1960)): to construct an object, one produces ‘an indicator for its basic state of affairs’ (§49), where such an indicator (‘Kennzeichen’) is a description, logically speaking a propositional function, which captures a necessary and sufficient condition for being that object. The ‘quasi object’ is then the class of all objects to which this description applies.

Consider the following passage from §158, in which Carnap illustrates that in a constructional system, individual concepts (in this case that of Luchs, a dog), just like general concepts, have classes, or classes of tuples as their extensions:

Let us use for clarification the following descending sequence of objects (or concepts): The dog (species) is a class to which my dog Luchs belongs. Luchs is a class whose elements are the “states” of Luchs. An individual state of Luchs (as a perceptual thing) is a class whose elements are points of the perceptual world. One such point is a many-place relation extension whose terms are four numerical terms (namely, the space-time coördinates) and one or more sense qualities; a sense quality is a class of “my experiences”. The latter are here envisaged as basic elements.

The name ‘Luchs’ refers to a quasi object, i.e. an extension of a concept (cf. §27ff), where this extension is a set which in turn contains further, logically constructed quasi objects (the states of Luchs), which themselves are logically constructed, and so on further down the chain of constructional definitions, until the most basic level, the level of the basic experiences of the constructing subject is reached. Since both the numerical structure of spacetime and sense qualities (quality classes) are constructed out of the basic relation *Rs* (see §§81, 112 and §125 respectively), we can ultimately translate any sentence containing the name ‘Luchs’ into sentences involving only (proper) predications of concepts to singular terms referring to basic experiences. The quoted passage informally sketches the crucial stages in Luchs’ construction and thereby gives us, in its third sentence, the means to (informally) formulate its constructional definition (see §35): the name ‘Luchs’ can in any sentence in which it occurs be replaced by a definite description picking out the mentioned class of states of Luchs, where Luchs is a perceptual objects in the sense of the Aufbau.

**Fig. 1:**
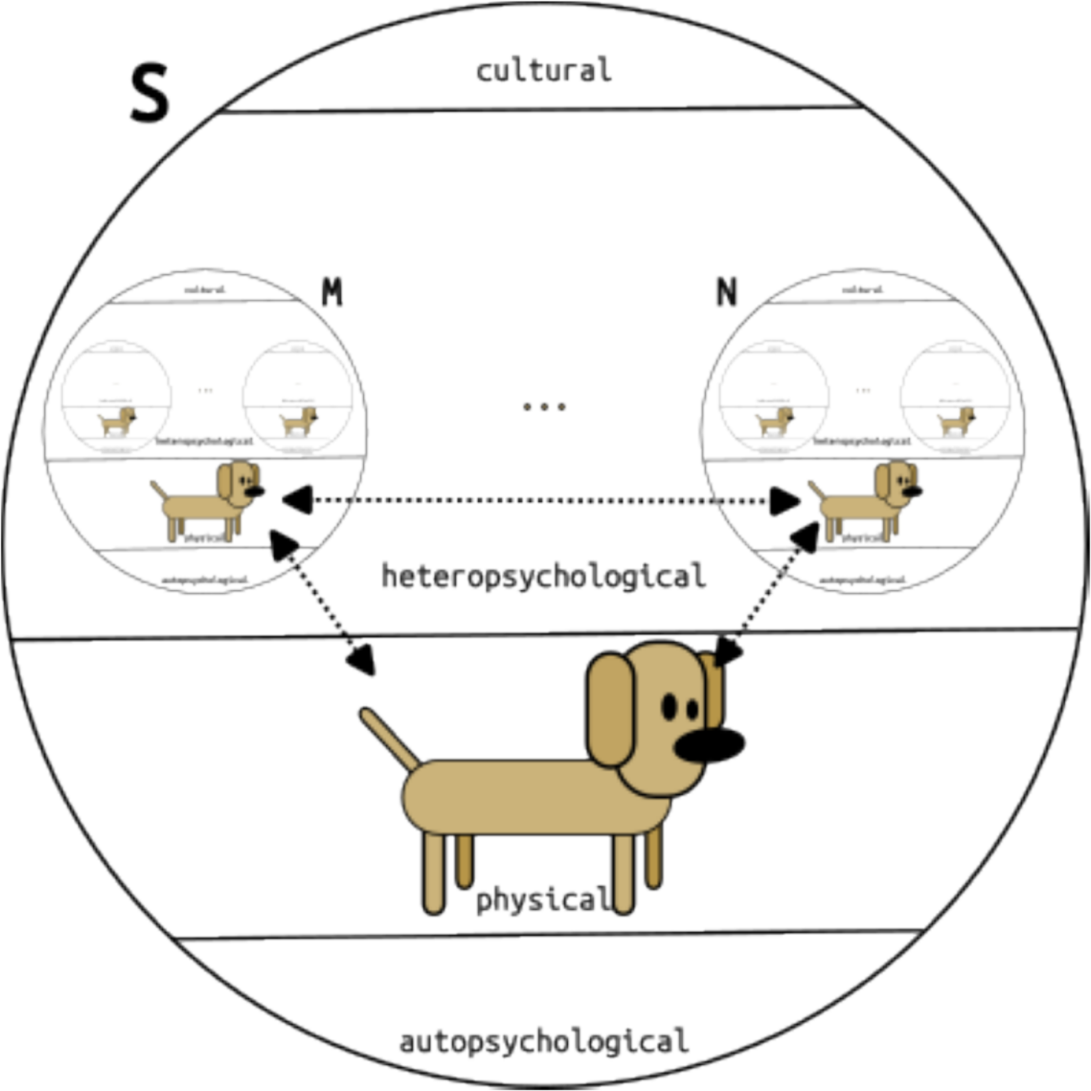
Intersubjective correspondence between Luchs on the physical levels of *S*’s constructional system and of two sub-systems constructed within *S*’s system for two other subjects *M* and *N*

The process (speaking metaphorically) of construction just illustrated is purely extensional:

By a *constructional definition* of the concept *a* on the basis of concepts *b* and *c*, we mean a rule of translation which gives a general indication how any propositional function in which *a* occurs may be transformed into a coextensive propositional function in which *a* no longer occurs, but only *b* and *c*. (§35.)

So as long as the Luchs-concept has the same extension as the concept corresponding to the set of states of the relevant perceptual thing, its constructional definition is admissible.

Since, as we have just seen, objects are identified with concepts (or rather concept extensions) and concepts are treated as propositional functions, functions from (quasi-)objects to truth-values, the fundamental notion of identity in the Aufbau, unlike in the context of Lewis’s modal metaphysics, cannot be that of objectual identity. Constructional theory instead by default relies on what Carnap calls logical identity:

Substitutability is the criterion for an identical nominatum: two designations are said to be synonymous if each propositional function which is turned into a true sentence through the substitution of one of the designations does the same upon the substitution of the other. *This is the definition of logical identity*. (§159.)

Logical identity in the sense just defined is nothing else than coextensionality, i.e. sameness of extension between propositional functions. (See §32.) Having clarified this, I can now address the remaining first guiding question of this section: what prevents the relata of the relation of intersubjective correspondence from being logically identical?^17^

The relation of intersubjective correspondence holds between pairs of objects which share all their physical properties, but belong to distinct constructional systems. As pointed out earlier in this subsection, the relevant constructional systems here are the main system and all of its sub-systems constructed for subjects other than the constructional subject. Figure 1 illustrates this for Carnap’s Luchs-example.

Pairs of other-systemly quasi objects on the physical level and above cannot be identical in the logical sense, because their constructional definitions, and consequently also the extensions with which Carnap identifies them, involve elements that are specific to the particular constructional (sub-)system to which they belong. This egocentricity prevents any physical object in a constructional system from standing in the relation of logical identity, the default notion of identity in the Aufbau, to any physical object in one of its sub-systems, and vice versa. This then settles the first question, the question of why intersubjectively corresponding object cannot instead simply be logically identical, i.e. identical in the standard sense of identity of the Aufbau.

### The theoretical role reading: verdict

This subsection identifies one respect in which Lewis’s counterpart relation is ‘very like’ Carnap’s relation of intersubjective correspondence: Both relations play the same role in the respective theories within which they occur, namely that of a replacement for the default notion of identity for objects which belong to distinct world-like entities. The counterpart relation replaces numerical identity for entities which are numerically distinct since they are part of distinct (real) possible worlds, but still have to be treated as, in a looser sense, being the same to give Lewis a simulacrum of trans-world identical objects. The relation of intersubjective correspondence replaces logical identity between quasi objects which are logically distinct, since their extensions differ relative to the constructional (sub-)systems to which they belong, but still have to be treated as, in a looser sense, being the same in order to give Carnap an objective notion of physical object. Read as a claim about this respect of similarity, or more specifically, a similarity claim which weighs this respect of similarity highly enough to render the two relations ‘very like’, Lewis’s claim in the footnote is hence true.

## Intermezzo: the placement of the footnote

Lewis’s footnote is a footnote to a passage which says that the counterpart relation is a relation of similarity which makes it ‘problematic in the way all relations of similarity are: it is the resultant of similarities and dissimilarities in a multitude of respects, weighted by the importances of the various respects and by the degrees of the similarities’ (Lewis (1968), p. 115.). Lewis does not spell out what these problems are, but he very likely had in mind^18^ that similarity relations are, due to the features which Lewis mentions, highly context-dependent (the weightings of different respects of similarity can change from context to context, depending on which of them are considered more important) and vague (for any *x*, there is typically no precise boundary between things which are overall similar and overall dissimilar to *x*).

Why did Lewis place the footnote here and not after what, in light of the reading I proposed in Section 3, seems like a better location, namely after the sentence ‘The counterpart relation is our substitute for identity between things in different worlds’ (Lewis (1968), p. 114)? Indeed, it is natural to take the placement of the footnote at the end of this passage to suggest that Lewis (mistakenly) thought that intersubjective correspondence is likewise a similarity relation and hence also subject to the same context-relative factors and the connected problems he mentions with respect to the counterpart relation. The problem is that, as shown in Section 2, intersubjective correspondence is an equivalence relation, which means that in virtue of its formal properties alone, it cannot be a similarity relation in Lewis’s sense.

Does the placement of the footnote hence reveal a substantial misunderstanding on Lewis’s part? Perhaps even a confusion of the relation of intersubjective correspondence with the basic relation of Carnap’s example system, *Rs* (recollection of similarity), which is indeed a relation of overall similarity? If so, then this would of course dispel any mystery surrounding the footnote and undermine the need for an in-depth discussion of it. Lewis’s claim of a likeness between the two relations would simply be wrong, end of the story. Should we attribute this rather striking mistake to Lewis?

To do so would be quite uncharitable. Lewis’s work on quasi-analysis in Lewis (1969) and his familiarity with Carnap’s introduction of a primitive notion of foundedness, which directly builds on the discussion of intersubjective correspondence in the preceding paragraphs, at least make it highly unlikely that he confused the two relations.

Fortunately, a more charitable interpretation is available: Given his heavy reliance on this particular notion of similarity throughout his works, Lewis likely intended the likeness claim to be read as a claim of *overall* similarity. Two relations can be overall similar, even to a high degree, without being similar in all respects, given that the specific respects in which they are similar carry a larger weight than those in which they are not. This interpretation is consistent with Lewis’s being fully aware that the two relations are not both similarity relations, i.e. do not share the same formal properties. It still attributes a mistake to him, namely the misleading placement of the footnote, but this mistake is certainly less egregious than that of confusing intersubjective correspondence with a similarity relation.

## Lewis the constructional theorist?

The availability of a true reading of the footnote lends further support to the claim that aspects of Lewis’s work in metaphysics stand in a relation of ancestry (in MacBride (2021), §9’s sense) to aspects of the Aufbau. The fact that he inserted the footnote and the availability of a coherent true reading of the similarity claim it contains suggest that Lewis saw a real and significant analogy between the two relations.

In this last section, I want to argue that the extent of motifs from the Aufbau which reoccur in Lewis’s philosophy goes beyond the ancestral relations between ideas pointed out in MacBride (2021) (in particular Lewis’s reliance on naturalness) and in this paper (his use of the counterpart relation as a replacement for identity). In particular, I want to argue that besides similarities in theoretical posits, there are also important methodological similarities. To make this point, I will focus on three examples: Lewis’s proposal to use quasi-analysis to define naturalness, his own retrospective framing of his reductive project in the philosophy of mathematics, and his reliance on similarity to analyse philosophical notions. Motifs from the Aufbau hence are more pervasive in Lewis’s philosophy than one might think and in particular extend to Lewis’s philosophical methodology. This raises a number of historical questions for future work, both about whether these motifs can be traced back through the work of Lewis’s teachers Quine and Goodman, who were both heavily influenced by Carnap, as well as about the extent to which they have been carried along in contemporary discussions which either depart from Lewis’s work, or were strongly influenced by his contributions.

### Quasi-analysis in ‘New Work for a Theory of Universals’

‘New Work for a Theory of Universals’ (Lewis, 1983) marked an important junction in Lewis’s philosophical development, since it saw him acknowledge that the nominalist, abundant view of properties he favoured had to be supplemented by a notion of naturalness. In the paper, Lewis considers two distinct approaches towards adding such a notion. MacBride (2021) has shown that the first approach, which adds naturalness as a non-logical primitive, can be linked to Carnap’s adoption of a primitive notion of foundedness in §154 of the Aufbau. The second proposal, which Lewis takes to lead into a form of resemblance nominalism, also points back to the Aufbau. It assumes that there are primitive resemblances between particulars and ‘define[s] natural properties in terms of the mutual resemblance of their members and the failure of resemblance between their members and their non-members’ (Lewis (1983), p. 347.). In this context, Lewis only explicitly refers to Goodman (1951); Quine (1969), and Morton (1975), but the second approach is nothing else than an application of (a more sophisticated version of) Carnapian quasi-analysis.^19^

### Lewis’s ‘construction’ of mathematics

In a passage from Lewis’s introduction to his ‘Collected Papers in Philosophical Logic’ (Lewis (1998), p. 3) Lewis explicitly discussed a connection he saw between the Aufbau and his own work in the philosophy of mathematics. According to Lewis, the last four papers of the collection (‘Policing the Aufbau’, ‘Finitude and Infinitude in the Atomic Calculus of Individuals’ (co-authored with Wilfrid Hodges), ‘Nominalistic set theory’, ‘Mathematics is Megethology’) ‘address questions arising in the construction of ambitious formalized philosophical systems’, of which the Aufbau ‘is perhaps the most ambitious’ (ibid.). Lewis’s discussion of how ‘Policing the Aufbau’ and the remaining three papers in the collection, which are concerned with the foundations of mathematics, fit into the overarching context of his works, suggests that he saw the latter three papers as a continuation in spirit of the reductive project of the Aufbau:

The Aufbau was built upon a framework of set theory, in much the way that modern mathematics normally is. But, mainly because of the set-theoretical paradoxes, it would be nice to replace set theory by mereology, the formal theory of the relation of part and whole. This is done, for instance, in Goodman’s *Structure of Appearance*. Still nicer would be to rebuild set theory itself by mereological means. In ‘Finitude and Infinitude in the Atomic Calculus of Individuals’, Hodges and I show an obstacle to this ambition: first-order mereology cannot express the difference between a finite and an infinite world. ‘Nominalistic Set Theory’ shows how to build approximate simulations of set theory within first-order mereology if we have a way to talk about the adjacency or non-adjacency of atoms. But if we give ourselves second-order mereology, ‘megethology’, we can do much better: we can exactly recapture standard set theory, and hence standard mathematics. In ‘Mathematics is Megethology’ I show how this can be done without falling into known paradoxes, and without allowing our framework to foist upon us any ontological commitments over and above those of the mathematics we set out to recapture.

One might think that this description of the project Lewis pursued in these papers, which, one may add, could have also included the predecessor to ‘Mathematics is Megethology’, Lewis’s short book *Parts of Classes*, does not sound much like that of the Aufbau. According to a view of the Aufbau established by Quine and Goodman in the 1950 s and, curiously, even accepted by Carnap himself in the preface to the Aufbau’s second edition written in 1961,^20^ the main ambition of the book was to put the whole of scientific knowledge on a phenomenalist basis. Indeed, if constructional theory is concerned only with a complete reduction of all of science, including in particular the empirical sciences, Lewis’s reduction of mathematics to megethology could hardly be mistaken for an exercise in constructional theory. Furthermore, it is relatively clear that Lewis did not see himself as reviving constructional theory. His description of ‘Policing the Aufbau’ in Lewis (1998), which echoes his two teachers Quine and Goodman in calling the Aufbau an ambitious project which ‘failed on its own terms’, (ibid) leaves little doubt about this.

However, in the Aufbau, Carnap seems to have, at least tentatively, entertained a more inclusive use of the label ‘constructional theory’, a use which would put Lewis’s project in its scope of application. In §35, Carnap calls Russell’s and Whitehead’s reduction of mathematics to logic, using scare quotes, a “constructional system”. As the use of scare quotes indicates, he was not entirely comfortable with classifying it as a genuine constructional system.

Why did Carnap not want to fully commit to assigning this label? The likely explanation can be found in §107:

[...]logical and mathematical objects are not actually objects in the sense of real objects (objects of the empirical sciences). Logic (including mathematics) consists solely of conventions concerning the use of symbols, and of tautologies on the basis of these conventions. Thus, the symbols of logic (and mathematics) do not designate objects, but merely serve as symbolic fixations of these conventions. Objects in the sense of real objects (including quasi objects) are only the basic relation(s) and the objects constructed therefrom.

The idea here is clear: the reduction of mathematics to logic is not in the scope of constructional theory. The latter is concerned only with the objects of the empirical sciences, the ‘real objects’, and mathematics (as well as logic) is concerned with highly conventional objects. Is this is a good reason to exclude mathematics from the scope of constructional theory? It seems that it is not.

First of all, according to Carnap, cultural objects of a highly conventional nature, such as the practice of hat-lifting (cf. §24), are in the scope of his outlined system. The conventional nature of logic which Carnap mentions can therefore hardly be a sufficient reason to exclude mathematical objects.

His reliance on the notion of reality does not help his cause either. First of all, the metaphysical notion, which might otherwise appear to be apt in this context, has no place in a constructional system (cf. §176). But Carnap also discusses a range of different notions of reality which are admissible, i.e. definable, in a constructional system, and these notions are also not fit to make Carnap’s argument work. The most salient of these is the notion of real-typical objects, a category to which non-temporal objects like numbers might in principle belong. The problem is that this notion is, in Carnap’s own words ‘a disjointed concept which is not clearly delineated’ and in ‘urgent need’ of a convention which settles the concept’s boundary (cf. §174). Absent such conventions, the category of real-typical objects is not clearly delineated, giving us no precise criterium for when an object could be classified as ‘real’ in this sense.

Finally, that Carnap has no scruples explicitly writing about the construction of the logical and mathematical objects in the beginning of §107 does not help his case either.

To sum up, Carnap was hesitant to outright classify Russell and Whitehead’s logicist project as an example of an application of constructional theory, however the reasons for Carnap’s hesitation do not seem to hold up. Since there is a clear similarity between that project and Lewis’s project of providing a megethological foundation for mathematics, it does seem that if the former can be considered a constructional project, the latter deserves the same sort of consideration.

### Similarity as a central building block

A further important methodological parallel between Lewis’s philosophy and the Aufbau concerns the use to which similarity is put in it. The basic relation *Rs* (*Recollection of Similarity*, see §78) of Carnap’s example system is a relation of overall similarity. Everything in the constructional system is, assuming for the sake of the argument a completed construction, ultimately definable in terms of it as the only non-logical primitive. Importantly for what I am about to say, this means that *Rs* is used to define a wide range of notions, such as a (preliminary) temporal order for the spacetime world (§120), physical objects like dogs (§158), and even cultural practices, such as hat-lifting (§24), which, at least overtly, have not much to do with the overall similarity between a person’s momentary experiences encoded in *Rs*.

Lewis was certainly aware of the use to which the relation was put in Carnap’s construction, as his work on quasi-analysis in Lewis (1969) demonstrates. The point I want to make now is that in certain parts of his philosophy, a relation of overall similarity plays a theoretical role which resembles that of *Rs* in the Aufbau in two key aspects. First, in that it is treated as a theoretical primitive, and second, that it is then used in defining overtly very different notions. Perhaps the most salient, but not the only, example of this is Lewis’s reliance on an ordering of possible worlds in terms of overall similarity in his semantics for counterfactual conditionals (see Lewis (1973a)).^21^

The core idea of this semantics is that a counterfactual conditional of the form ‘If *P* were the case, then *Q* would be the case’ is true if *Q* is true in the closest possible worlds in which *P* is true. The crucial ingredient which permits this semantics to specifically account for counterfactuals, rather than strict conditionals (conditionals of the form ‘It is necessarily the case that, if *P*, then *Q*’), is the notion of closeness. This notions refers us to an ordering of possible worlds by their comparative overall similarity to the actual world, or to use Lewis’s spatial metaphor, a concentric system of spheres of overall similarity centred on the actual world. (See Lewis (1973a), §1.3.)

We need not go into more details to highlight the two respects in which the role of this similarity ordering in Lewis’s semantics resembles that of *Rs* in the sketched constructional system from part IV of the Aufbau. First, the similarity ordering is primitive, that is, not further analysable in other terms. (See Lewis (1973a), §4.2.)^22^ Second, besides merely fulfilling its manifest purpose of providing truth conditions for sentences involving counterfactual conditionals, the semantics plays an important further role in Lewis’s philosophy: Lewis crucially relies on counterfactuals, and in particular on his own semantic analysis of them in order to analyse a number of, at least prima facie unrelated, philosophical notions and ideas. Three important examples are his (to this day highly influential) counterfactual analysis of causation (Lewis, 1973c), his account of the apparent openness of the future (Lewis, 1979a), and his reinforcement of the conditional analysis of dispositions (Lewis, 1997a).

Now, one might object that the analogy between the roles which a relation of overall similarity plays in these examples and in the Aufbau is not perfect. That the respective relation is used as a theoretical primitive in both contexts is beyond dispute, but let me mention two overt dissimilarities regarding the uses to which it is put. First, in the Aufbau, the relation *Rs* is at the bottom of a reductive hierarchical system of definitions which is built up in such a way that every notion occurring in it can ultimately be *defined* in terms of *Rs* (plus logical notions). The ordering of possible worlds in terms of comparative overall similarity involved in Lewis’s semantics is in contrast not at the bottom of a comparable hierarchy of definition. In particular, the two ‘definitional’ relations between overall similarity and the counterfactual conditional, and between the latter and causation are not the same; Lewis uses overall similarity to *recursively define truth conditions* for sentences involving counterfactuals, but proposes a *conceptual analysis* of causation which relies on counterfactuals. Second, Carnap explicitly placed a number of metaphysical notions, including in particular causation (§165) outside the scope of his constructional system, which of course means that such notions are not definable in terms of *Rs*. Lewis in contrast relies on the overall similarity ordering of possible worlds to analyse both causation, as well as other philosophical notions which Carnap would have dismissed as metaphysical and unscientific and hence unfit to be accommodated within a constructional system.

These are genuine differences, but they do not break the analogy which I intend the examples to illustrate. Let me begin with the first point, that Lewis’s overall similarity ordering is not at the basis of an Aufbau-style hierarchy of definitions. While this is in one sense true, a closer look at both Lewis’s systematic philosophy and at the structure of the Aufbau brings out another aspect of the analogy which remains untouched. Lewis was not engaged in a project of devising a ‘grand theory of everything’, a label which can perhaps rightfully applied to Carnap’s work in the Aufbau, but there is no denying that over his career, he built a philosophical system of another sort.^23^ His system is rather an integrated package of modular doctrines, which allows one to take a relatively homogenous approach to a wide range of philosophical topics. (See Beebee and Fisher (2022), pp. 7f.) Lewis’s counterfactual analysis of causation (or his counterpart theory, or his modal realism) can hence be said to fit into a malleable, but still holistic structure delineated by this integrated package. There is of course a certain disanalogy between this holistic structure and the strictly foundationalist structure of Carnap’s example system, but a striking parallel in methodology remains: Both Carnap in the Aufbau, as well as Lewis over most of his career worked with a fixed, explicitly introduced set of concepts and formal methods, partly of their own design, which gave them a framework into which concepts and ideas they encountered in their investigations were then tried to fit.

To address the second point: while Carnap does reject the idea of there being a ‘real’ relation of causation, a relation of ‘bringing about’ which holds between events, he does at the same time offer a (in his eyes) scientifically acceptable replacement for such a notion, namely causal laws. He takes such laws to be process laws with temporal proximity, where laws of this kind are, in Humean fashion, simply identified with sentences stating functional dependencies between assignments of qualities to world points on the physical level of a constructional system which are at a close temporal distance. So as far as Carnap is concerned, if someone really wanted to speak about causation, they would have a constructional system-intrinsic means to do so at their disposal. (See §165.) Generous offers of the same sort are even extended to those who want to use even more radical metaphysical notions such as identity (§159), essence (§161), or the self (§163). In making these offers, Carnap basically takes the same methodological approach Lewis would later take, namely that of using a particular conceptual toolkit drawn from a philosophical system to provide the best conceptual approximations of what would else be vague or obscure notions. To be sure, Lewis does not first explicitly dismiss the philosophical notions he analyses as obscure as Carnap does, but unlike him, Lewis was under no pressure to provide a justification for his approach. The method of explicitly re-defining traditional philosophical notions using logical and conceptual means in order to make them more tractable was already a well-established practice in the philosophical culture in which he worked. It is hard not to see the Aufbau as a contributing cause in the development of this culture.

## Footnotes

1 It highlights an important motif in Lewis’s philosophy. Compare, for example, the discussion of the ordering of possible worlds in spheres of overall similarity around the actual world in Lewis (1973a), Section 1.3. See also Lewis (1973b) and Lewis (1979a), and see Kroedel and Huber (2013); Morreau (2010) for recent discussions drawing on analogue problems with preference orderings in social choice theory.

2 The particular placement of the footnote will be discussed in Section 4.

3 See in particular his work on the ‘Ramsey–Carnap–Lewis’ analysis of theoretical terms’ Lewis (1970, 1972, 1997b), or Lewis (2009). Lewis also discussed Carnap’s work on inductive Logic in Lewis (1971).

4 Lewis (1969). See Carnap (1969), §72 for Carnap’s statement, Goodman (1951), V.3.70 for an influential statement of the impact of the problem on the project of the Aufbau, and Leitgeb (2007, 2011) and Mormann (2009) for discussions.

5 More exactly, the axioms of counterpart theory prescribe that objects can exist in at most one possible world (postulate P2), that the only counterpart an object has in its own world is itself (postulate P5), and that anything in a world is its own counterpart (postulate P6). See Lewis (1968), p. 114.

6 Carnap (1928/1998); I will cite the English translation (Carnap, 1969) by Rolf A. George throughout the paper which translates ‘Konstitutionssystem’ as ‘constructional system’.

7 See the preface to the first edition of the Aufbau, Carnap (1969), p. xvii, for a clear statement of the anti-metaphysical agenda in the Aufbau. For a comparative discussion of Carnap’s critiques of metaphysics in the Aufbau and in Carnap (1959), see Declos (2017), Section 2.1.5, pp. 131ff. See also Friedman (2007).

8 See MacBride (2021), p. 27.

9 For a different perspective on the relation between the Aufbau and Lewis’s philosophy, see the discussion of what a second Aufbau drawing on Lewis’s project of Humean Supervenience might have looked like in Chalmers (2022).

10 See MacBride (2021), p. 8 and https://findingaids.princeton.edu/catalog/C1520_c574.

11 See Carnap (1969), §34 and the first paragraph of Lewis (1968).

12 See Carnap’s distinction between four languages which can be used to describe a constructional system in §95.

13 Lewis only refers to ‘avoidable problems of individuation’ to motivate the postulate in Lewis (1968), footnote 2, p. 114, but later elaborates the problem of accidental intrinsics in Lewis (1986), pp. 199-201.

14 Lewis (1968), p. 114. This assumption is built into Lewis’s axiomatization of counterparts theory via postulates P1: $\forall x \forall y (Ixy \rightarrow Wy)$ (things can only be in worlds) and P2: $\forall x \forall y \forall z ((Ixy \wedge Ixz) \rightarrow y=z)$ (nothing can be in more than one world), where ‘*Ixy*’ stands for ‘*x* is in *y*’ and ‘*Wx*’ for ‘*x* is a (possible) world’.

15 Cf. Lewis (1968), p. 114.

16 That is, because even though the assignment of colours and other qualities to world points proceeds from a subjective perspective, it is supposed to also cover things not directly perceived by the subject by extrapolation (see desiderata 7ff in §§126-127 and §135) and, at a later stage in the construction, is supposed to be extended and corrected through observation of the expressions of other subjects (see §138) and their testimony (see §142).

17 A range of other relations which either bear identity in their name, or are in other respects similar to numerical identity occur in the Aufbau. In his explicit discussion of identity in §159, Carnap singles out two relations which are ‘frequently confused with (proper) identity’, namely intersubjective correspondence and genidentity, the relation between any two world points which are on the same world line, or between any two states of the same visual thing (see §§128, 159). A third identity-like relation is the relation of part identity between elementary experiences defined in §113. As Moulines (1991), p. 276 points out, this latter relation can, despite what one might initially think, not be used to define numerical identity between experiences in terms of part identity with respect to all parts of two elementary experiences: Elementary experiences are not repeatable, so even two qualitatively identical experiences, i.e. experiences which are part-identical with respect to all their parts, will still be distinct.

18 This conjecture is supported by Lewis’s later discussion of overall similarity in Lewis (1979a).

19 See Leitgeb and Carus (2025), supplement D, §3. Note that, at this point in time, Lewis did not take the well-known difficulties with quasi-analysis to be a decisive strike against the second approach. He however seems to soon have changed his mind, favouring the first approach. See Lewis (1983), p. 348 and Lewis (1986), pp. 63ff, where he writes that the quasi-analytic approach to naturalness, again only indicated via a reference to Goodman (1951), runs into ‘serious trouble’.

20 See Carnap (1969), p. vi. What makes Carnap’s acceptance of this reading seem curious is his explicit commitment to presenting constructional theory as a general methodology and his discussion of possible constructional systems with other bases in different passages in the Aufbau. Compare Pincock’s similar remark (Pincock (2009), p. 956) about Carnap’s later acceptance of Goodman’s and Quine’s criticisms of the Aufbau in his contributions to Schilpp (1963).

21 Overall similarity plays a similar theoretical role in counterpart theory (see Section 3.1), but see also Lewis’s account of truth in fiction (Lewis, 1978) and his discussion of commands in Lewis (1979b).

22 It is interesting to note that when pressed to say more about the ordering in the context of his response to Fine’s future similarity objection, Lewis gives a list of four ranked weights and priorities which the ordering has to satisfy in order for his semantics to assign the expected truth-value to the relevant counterfactual conditional. This list and Lewis’s remark that ‘[p]lenty of unresolved vagueness remains, of course, even after we have distinguished the four sorts of respect of comparison and ranked them in decreasing order of importance’, (Lewis (1979a), p. 472)) brings to mind, perhaps only accidentally, Carnap’s list of desiderata for the assignment of colours to world points from §126 and his remark, that they ‘cannot be precisely satisfied’ (ibid).

23 Lewis’s own contradictory statements have led to some confusion about whether one should think of Lewis’s philosophy as a philosophical system. This confusion is cleared up by Beebee and Fisher (2022), §3, and I rely on their disambiguation of his statements here.

## References

[CR1] Beebee, H., & Fisher, A. R. J., (2022). Introduction. In H. Beebee, & A. R. J. Fisher (Eds.), *Perspectives on the Philosophy of David K. Lewis, chapter 1* (pp. 1–12). Oxford University Press.

[CR2] Carnap, R. (1928/1998). Der logische Aufbau der Welt. In F. M. Verlag (Ed.), (3rd ed.) .

[CR3] Carnap, R. (1959). The elimination of metaphysics through logical analysis of language. In A. J. Ayer (Ed.), *Logical Positivism*, The Library of Philosophical Movements, (pp. 60–81). Free Press. Translated by Arthur Pap; originally published as ‘Überwindung der Metaphysik durch logische Analyse der Sprache’ in *Erkenntnis*, (vol. 2(1931), nr. 1, pp. 219–41).

[CR4] Carnap, R. (1969). The logical structure of the world and pseudoproblems in philosophy. University of California Press. Translated by Rolf A. George.

[CR5] Chalmers, D. J. (2022). Carnap’s second Aufbau and David Lewis’s Aufbau. In H. Beebee, & A. R. J. Fisher (Eds.), *Perspectives on the Philosophy of David K. Lewis* (chap. 6, pp. 92–117). Oxford University Press.

[CR6] Declos, A. (2017). La métaphysique de Nelson Goodman. PhD thesis, Université de Ottawa and Université de Lorraine.

[CR7] Frege, G. (1960). Function and concept. In P. Geach, & M. Black (Eds.), *Translations from the Philosophical Writings of Gottlob Frege* (2nd ed., pp. 21–41). Basil Blackwell.

[CR8] Friedman, M. (2007). The *Aufbau* and the rejection of metaphysics. In M. Friedman, & R. Creath (Eds.), *The Cambridge Companion to Carnap* (pp. 129–152). Cambridge University Press.

[CR9] Goodman, N. (1951). The structure of appearance. Harvard University Press.

[CR10] Kroedel, T., & Huber, F. (2013). Counterfactual dependence and arrow. *Noûs,**47*(3), 453–466.

[CR11] Leitgeb, H. (2011). New life for Carnap’s “Aufbau?” *Synthese, **180*(2), 265–299.

[CR12] Leitgeb, H., & Carus, A. (2025). Rudolf Carnap. In E. N. Zalta, & U. Nodelman (Eds.), The Stanford Encyclopedia of Philosophy. Metaphysics Research Lab, Stanford University, Fall 2025 edition.

[CR13] Leitgeb, H. (2007). A new analysis of quasianalysis. *Journal of Philosophical Logic,**36*(2), 181–226.

[CR14] Lewis, D. (1969). Policing the Aufbau. *Philosophical Studies, 20*, 13–17. Reprinted in *Papers in philosophical logic*.

[CR15] Lewis, D. (1973a). Counterfactuals. Blackwell Publishing.

[CR16] Lewis, D. K. (1979). A problem about permission. In E. Saarinen, R. Hilpinen, I. Niiniluoto, & M. Provence (Eds.), *Essays in Honour of Jaakko Hintikka on the Occasion of His Fiftieth Birthday on January 12, 1979* (pp. 163–175). Reidel.

[CR17] Lewis, D. (1986). On the plurality of worlds. Blackwell Publishing.

[CR18] Lewis, D. (1998). Introduction. In *Papers in philosophical logic* (pp. 1–4). Cambridge University Press.

[CR19] Lewis, D. (2009). Ramseyan humility. In D. Braddon-Mitchell, & R. Nola (Eds.), *Conceptual Analysis and Philosophical Naturalism* (pp. 203–222). MIT Press.

[CR20] Lewis, D. K. (2020). Philosophical Letters of David K. Lewis Volume 1: Causation, Modality, Ontology. Oxford University Press.

[CR21] Lewis, D. K. (1968). Counterpart theory and quantified modal logic. *Journal of Philosophy,**65*(5), 113–26.

[CR22] Lewis, D. (1970). How to define theoretical terms. *Journal of Philosophy,**67*(13), 78–95.

[CR23] Lewis, D. (1971). Immodest inductive methods. *Philosophy of Science,**38*(1), 54–63. 10.1086/288339

[CR24] Lewis, D. (1972). Psychophysical and theoretical identifications. *Australasian Journal of Philosophy,**50*(3), 249–258.

[CR25] Lewis, D. (1973). Counterfactuals and comparative possibility. *Journal of Philosophical Logic,**2*(4), 418–46.

[CR26] Lewis, D. (1973). Causation. *Journal of Philosophy,**70*(17), 556–567. 10.2307/2025310

[CR27] Lewis, D. K. (1978). Truth in fiction. *American Philosophical Quarterly,**15*(1), 37–46.

[CR28] Lewis, D. (1979). Counterfactual dependence and time’s arrow. *Noûs,**13*(4), 455–476.

[CR29] Lewis, D. (1983). New work for a theory of universals. *Australasian Journal of Philosophy,**61*(4), 343–77.

[CR30] Lewis, D. (1997a). *Finkish dispositions. Philosophical Quarterly,**47*, 143–58.

[CR31] Lewis, D. (1997b). Naming the colours. *Australasian Journal of Philosophy,**75*(3), 325–42.

[CR32] MacBride, F. (2021). Rudolf Carnap and David Lewis on metaphysics. *Journal for the History of Analytical Philosophy, 9*(1).

[CR33] Mormann, T. (2009). New work for Carnap’s quasi-analysis. *Journal of Philosophical Logic,**38*(3), 249–282.

[CR34] Morreau, M. (2010). It simply does not add up: Trouble with overall similarity. *Journal of Philosophy,**107*(9), 469–490.

[CR35] Morton, A. (1975). Complex individuals and multigrade relations. *Noûs,**9*(3), 309–318.

[CR36] Pincock, C. (2009). Carnap’s logical structure of the world. *Philosophy Compass,**4*(6), 951–961.

[CR37] Quine, W. V. O. (1969). Natural kinds. In *Ontological Relativity and Other Essays* (pp. 114–138). Columbia University Press.

[CR38] Schilpp, P. A. (1963). The Philosophy of Rudolf Carnap, volume 11 of The Library of Living Philosophers. Open Court.

[CR39] Ulises Moulines, C. (1991). Making sense of Carnap’s “Aufbau”. *Erkenntnis, 35*(1-3), 263–286.

